# A cost comparison of electronic and hybrid data collection systems in Ontario during pandemic and seasonal influenza vaccination campaigns

**DOI:** 10.1186/1472-6963-11-210

**Published:** 2011-09-01

**Authors:** Jennifer A Pereira, Julie Foisy, Jeffrey C Kwong, Christine L Heidebrecht, Susan Quach, Sherman D Quan, Maryse Guay, Beate Sander

**Affiliations:** 1 Department of Surveillance and Epidemiology, Public Health Ontario, Toronto, Canada; 2 Institute for Clinical Evaluative Sciences, Toronto, Canada; 3 University Health Network, Toronto, Canada; 4 Département des sciences de la santé communautaire, Université de Sherbrooke, Longueuil, Canada

## Abstract

**Background:**

During the pandemic (H1N1) 2009 influenza vaccination campaign, health regions in Canada collected client-level immunization data using fully electronic or hybrid systems, with the latter comprising both electronic and paper-based elements. The objective of our evaluation was to compare projected five-year costs associated with implementing these systems in Ontario public health units (PHUs) during pandemic and seasonal influenza vaccination campaigns.

**Methods:**

Six PHUs provided equipment and staffing costs during the pandemic (H1N1) 2009 influenza vaccination campaign and staffing algorithms for seasonal campaigns. We standardized resources to population sizes 100,000, 500,000 and 1,000,000, assuming equipment lifetime of five years and public health vaccine administration rates of 18% and 2.5% for H1N1 and seasonal campaigns, respectively. Two scenarios were considered: Year 1 pandemic and Year 1 seasonal campaigns, each followed by four regular influenza seasons. Costs were discounted at 5%.

**Results:**

Assuming a Year 1 pandemic, the five-year costs per capita for the electronic system decrease as PHU population size increases, becoming increasingly less costly than hybrid systems ($4.33 vs. $4.34 [100,000], $4.17 vs. $4.34 [500,000], $4.12 vs. $4.34 [1,000, 000]). The same trend is observed for the scenario reflecting five seasonal campaigns, with the electronic system being less expensive per capita than the hybrid system for all population sizes ($1.93 vs. $1.95 [100,000], $1.91 vs. $1.94 [500,000], $1.87 vs. $1.94 [1,000, 000]). Sensitivity analyses identified factors related to nurse hours as affecting the direction and magnitude of the results.

**Conclusions:**

Five-year cost projections for electronic systems were comparable or less expensive than for hybrid systems, at all PHU population sizes. An intangible benefit of the electronic system is having data rapidly available for reporting.

## Background

Mass immunization clinics are often employed by public health organizations when a substantial proportion of the population needs to be vaccinated quickly, such as for seasonal influenza vaccination campaigns or pandemic emergencies. Planning for these high-volume clinics includes consideration of the approach that will be used to collect client-level vaccination data to help ensure optimal vaccine delivery processes, and timely assessment of vaccine coverage and surveillance statistics [1].

In preparation for the pandemic (H1N1) 2009 influenza vaccination campaign, every Canadian province/territory mandated data reporting requirements for their jurisdictions. While many health regions collected these immunization data using pre-existing approaches, other jurisdictions adopted new methods. Ontario was the only province in which fully electronic influenza immunization systems were used. Thirty of Ontario's thirty-six public health units (PHUs) collected H1N1 immunization data using this type of system, whereby client demographic, medical history and vaccination administration data were immediately inputted electronically, facilitating their use for program planning and reporting. Hybrid systems were implemented across Ontario's remaining PHUs, comprised of mainly paper-based data collection, with use of an electronic component at client registration or during post-vaccination transfer of data from paper.

In a qualitative study involving interviews with pandemic planners across Canada prior to the vaccination campaign, cost was identified as a primary perceived barrier to implementing an electronic system to collect client-level immunization data [2]. To provide valuable information to decision-makers planning mass vaccination campaigns, our study objective was to compare the costs to Ontario PHUs associated with implementing electronic and hybrid immunization data collection systems, during both pandemic and seasonal influenza vaccination campaigns.

## Methods

### Analysis

We performed a costing analysis comparing electronic and hybrid approaches to immunization data collection used in Ontario during the H1N1 vaccination campaign, from the perspective of the PHU; only direct equipment and staffing costs were included.

#### Electronic System

The fully electronic immunization data collection system considered in this study is the Protocol for Electronic Clinic Systems (PECS), developed by Niagara Region, Ontario, Canada. Clients register with a clerk equipped with a laptop who records their demographic information (by swiping their driver's license or health card) and medical history data. An immunization nurse then retrieves each individual's record from the networked database, reviews medical history before administering the vaccine, and enters data regarding the vaccine given (injection site, vaccine name/lot number/expiry date) into the system. Proof of vaccine is printed directly from the client record and provided to each client before they exit the clinic. The individual vaccine records created at each immunization clinic are uploaded into a regional database from which reports are produced.

#### Hybrid System

The hybrid system considered in this study combines both electronic and paper-based components. At registration, all client demographic and medical history data are recorded on paper by the client and confirmed by the clerk. After the immunization nurse reviews the medical history and administers the vaccine, the nurse writes out a paper record of the vaccination information as well as a proof of vaccine and provides the latter to the client. All client data are later inputted into an electronic database by data entry clerks.

Given that a five-year time-frame includes equipment purchase and utilization until replacement is required, the primary outcome was overall five-year costs in 2009 Canadian dollars, per data collection system. Reported results have been discounted at 5% [3].

### Model

We developed a provincial model in Microsoft Excel to compare the equipment and staffing costs associated with implementing data collection systems during influenza vaccination campaigns. Two scenarios were tested: in Scenario 1, Year 1 was modeled as a pandemic campaign directly based on the costing data provided, while Years 2-5 were assumed to be seasonal influenza vaccination campaigns; in Scenario 2, all five years were modeled as seasonal campaigns. It was assumed that the system would be implemented for the first time in a given jurisdiction in Year 1 (requiring purchase of equipment), electronic equipment would be replaced every five years only, and that costs for Years 2-5 would therefore be mainly for support and maintenance. The model predicted the total five-year costs as well as the cost per capita per PHU associated with both systems in each scenario.

The model was based on costing and resource utilization data received from participating Ontario PHUs, and standardized for populations of 100,000 (small), 500,000 (medium), and 1,000,000 (large).

### Data

Parameters describing: i) the costs associated with the data collection systems employed during the pandemic (H1N1) 2009 vaccination campaign; and ii) comparative resource use during seasonal influenza campaigns, were collected through questionnaires and telephone interviews with managers and information technology (IT) staff from a convenience sample of six Ontario PHUs that participated in a related efficiency study [4]. Each of these PHUs utilized a fully electronic system for the pandemic campaign, but had used paper-based or hybrid data collection systems for recent seasonal campaigns.

Equipment and salary costs were compiled (Table 1). The base case consisted of mid-range cost values for all parameters, unless otherwise stated. Equipment costs were mainly associated with the electronic system, although laptops were also required for post-vaccination data entry for the hybrid system. Software fees for the electronic system were included in the analysis; however, because they were provided by the vendor on the condition that they would not be divulged, we also conducted a threshold analysis to determine the total five-year software cost at which the hybrid and electronic systems costs were equal.

**Table 1:** Equipment/Staffing Costs*

Equipment	Unit Cost	Unit Cost Range	Expected Lifetime
Server	$1,300	$1,052 - $1,549	5 years
Router	$173	$76 - $269	5 years
Switches	$323	N/A	5 years
Laptop	$1,144	$747 - $1,540	5 years
Printer	$874	$236 - $1,511	5 years
Cables	$134	N/A	5 years
Printing**	$6,230^-^	$3,000 - $9,460	Annual cost
Miscellaneous items***	$10,869^-^	$10,075 - $11,663	Annual cost^°^

**Staff**	**Hourly Wage**	**Hourly Wage Range**	

Nurse	$46^~^	$32 - $50	
IT personnel	$40	$40	
Registration/data entry clerk	$23.75	$22 - $25.50	

* Source: Convenience sample of Ontario PHUs (n = 6)

** Includes carbon copies, consent forms, vaccine administration forms, proof of vaccination printing, toner etc.

^- ^Listed costs are total annual costs, rather than unit costs.

*** Includes magnetic swipe-card readers, laptop cases, air cards, battery packs, USB keys, storage cases, laptop locks etc.

^°^Only a portion of H1N1 miscellaneous equipment costs is expected to be incurred during seasonal campaigns.

^~ ^Base case wage was chosen because it was consistently reported by the sources.

Assumptions for Scenario 2 and Years 2-5 of Scenario 1 were derived based on input from the participating PHUs who estimated their immunization data collection resource use during previous seasonal campaigns relative to the pandemic campaign (Table 2). Since a smaller proportion of a PHU's population is vaccinated by public health during seasonal campaigns compared to a pandemic campaign, less equipment and staffing resources are required. The proportion of H1N1 staffing hours used during seasonal campaigns (15%) was chosen based on: i) input from three PHUs in the convenience sample; and ii) the ratio of assumed H1N1 public health vaccination rate (18%) to the assumed seasonal influenza public health vaccination rate (2.5%) (unpublished data).

**Table 2:** Model Assumptions

Parameter	*Seasonal Influenza Vaccination Campaign *Assumptions	Source
Coverage Rate*	2.5%	Ontario PHUs
Paper costs	1/3 of costs during H1N1 campaign	Ontario PHUs
Miscellaneous costs	1/3 of costs during H1N1 campaign	Ontario PHUs
Nursing hours	15% of hours during H1N1 campaign	Ontario PHUs
Clerk hours	15% of hours during H1N1 campaign	Ontario PHUs
IT hours	15% of hours during H1N1 campaign	Ontario PHUs
Nurse training hours**	15% of hours during H1N1 campaign	Ontario PHUs
IT training hours**	15% of hours during H1N1 campaign	Ontario PHUs

**Parameter**	***Hybrid System *Assumptions**	**Source**

Laptop (n)	1 laptop per 10,000 vaccinees	Unpublished data
Paper costs	200% of electronic system	Assumption
Miscellaneous costs	10% of electronic system	Assumption
Nursing hours	133% of hours needed for electronic system	Ontario PHUs
Clerk hours	Equal to electronic system	Ontario PHUs
IT hours	None required	Ontario PHUs
Data sorting time	7 hours per 500 records	Billittier *et al*. [6]
Post-clinic data entry hours	62 seconds per vaccine record	Quach *et al*. [5]

*Percent of PHU population vaccinated by public health

** The model assumes that all IT and nursing staff involved with a PHU's mass vaccination campaign will be trained annually.

Assumptions for the hybrid system resource use in comparison to the electronic system were based on estimates from the convenience sample of Ontario PHUs as well as peer-reviewed literature (Table 2). Given that the majority of miscellaneous costs are equipment-related, and that hybrid systems use minimal equipment, we assumed that the miscellaneous cost of the hybrid system would be 10% that of the electronic system. Similarly, as the hybrid system utilizes paper forms for the majority of data collection tasks, whereas only the proof of vaccination administration is printed on paper for the electronic system, we conservatively assumed that the paper costs of the hybrid system would be twice that of the electronic system.

It was assumed that post-vaccination data entry for the hybrid system would require one laptop per 10,000 clients vaccinated, based on interviews with public health contacts in a separate province (unpublished data). The assumption that the hybrid system would require one-third more nurse hours than the electronic system was based on staffing formulas provided by two of the PHUs, indicating that a nurse could see 20-30 clients per hour when collecting data using a hybrid system, compared to over 40 clients per hour with an electronic system. Additionally, in a sub-analysis of data from our time and motion study examining immunization data collection during the pandemic vaccination campaign in Canada [5], we compared nursing time per client for PHUs implementing PECS versus organizations that: i) used a hybrid system, and ii) collected a similar number of data elements from clients as PECS users; we found that nursing time per client for the organizations using the electronic system was almost 40% shorter than for jurisdictions using a hybrid system. For the model, we chose to use the conservative 33% value as the base case, and to test a larger range in the sensitivity analysis.

The duties of the registration clerks were considered when developing related assumptions: for the electronic system, the clerk uses swipe-card technology to populate the client's demographic data fields and manually enters other necessary information, while for the hybrid system, the clerk either completes the client's form or reviews what the client has completed before they proceed to the immunization nurse. It was therefore expected that the systems would require an equal number of registration clerk hours.

Compared to the electronic system, the hybrid system requires resources for two additional data processing steps: counting and sorting client forms, and subsequent data entry into an electronic database. Billittier *et al*. found that seven person-hours were required to sort and alphabetize paper forms for 500 influenza vaccinations; this value was used in our model [6]. We calculated expected hours for data entry clerks by multiplying the number of vaccinated individuals within a PHU (public health administration rate × population size) with time required to enter one client's record; this was assumed to be 62 seconds based on the results of our time and motion study [5].

IT support was assumed to be required for the electronic system only and included time spent setting up the electronic database, pre-testing the system and providing clinic support.

### Sensitivity Analysis

Data uncertainty and jurisdictional variation in parameters were explored with one-way sensitivity analyses. Several clinic parameters (number of nurses, number of IT personnel, etc.) and economic parameters (equipment costs, salary wages, etc.) were modified within a pre-specified range based on data from public health contacts, published literature and best/worst case assumptions.

### Research Ethics Board Approval

This study received approval from the Research Ethics Board at the University of Toronto, Toronto, Canada.

## Results

### Base Case

*Scenario 1 (1^st ^year pandemic + 4 years of seasonal influenza vaccination campaigns): *Assuming a pandemic in Year 1 only, the five-year costs per capita for the electronic system is less than the hybrid system for all PHU population sizes: $4.33 vs. $4.34 [100,000], $4.17 vs. $4.34 [500,000], $4.12 vs. $4.34 [1,000,000]. As the electronic system's software fees are based on a sliding scale that is dependent on PHU size, there is a decrease in cost per capita with larger PHUs. Of total costs, staffing comprises 83-87% for electronic systems and > 98% for hybrid systems (Table 3).

**Table 3:** Total Five-Year Cost Comparison of Electronic and Hybrid Immunization Data Collection Systems for Scenario 1 (1^st ^year pandemic + 4 years of seasonal influenza vaccination campaigns)*

	*PHU population = 100,000*	*PHU population = 100,000*	*PHU population = 500,000*	*PHU population = 500,000*	*PHU population = 1,000,000*	*PHU population = 1,000,000*
	Electronic	Hybrid	Electronic	Hybrid	Electronic	Hybrid
**Equipment Costs** ***Equipment Costs***^***u***^	$75,200 *$77,377*	$7,405 *$7,774*	$296,907 *$306,887*	$37,024 *$38,868*	$547,447 *$563,775*	$74,049 $77,735

**Staffing Costs** ***Staffing Costs***^***u***^	$357,546 *$373,442*	$426,817 $*445,773*	$1,787,728 *$1,867,210*	$2,134,084 *$2,228,867*	$3,575,456 *$3,734,419*	$4,268,170 *$4,457,734*

**TOTAL COSTS** **(per capita costs)** ***TOTAL COSTS***^***u***^ ***(per capita costs)^u^***	$432,746 ($4.33) *$450,819* *($4.51)*	$434,222 ($4.34) *$453,547* *($4.54)*	$2,084,635 ($4.17) *$2,174,097* *($4.35)*	$2,171,108 ($4.34) *$2,267,735* *($4.54)*	$4,122,903 ($4.12) *$4,298,194* *($4.30)*	$4,342,218 ($4.34) *$4,535,469* *($4.54)*

**System Cost Difference** ***System Cost Difference***^***u***^		**$1,476** ***$2,728***		**$86,473** ***$93,368***		**$219,315** ***$237,275***

*Total costs have been discounted at 5%.

^u ^= Undiscounted

*Scenario 2 (5 years of seasonal influenza vaccination campaigns): *For the scenario reflecting five regular influenza seasons, the five-year costs per capita of the electronic system are also less than the hybrid system for all population sizes. Similar to Scenario 1, the between-system cost difference increases as the population size increases ($1.93 vs. $1.95 [100,000], $1.91 vs. $1.94 [500,000], $1.87 vs. $1.94 [1,000,000]). For PHUs using fully electronic systems, staffing-related expenses comprise 83-85% of electronic system total costs and > 98% of hybrid system total costs (Table 4).

**Table 4:** Total Five-Year Cost Comparison of Electronic and Hybrid Immunization Data Collection Systems for Scenario 2 (5 years of seasonal influenza vaccination campaigns)*

	*PHU population = 100,000*	*PHU population = 100,000*	*PHU population = 500,000*	*PHU population = 500,000*	*PHU population = 1,000,000*	*PHU population = 1,000,000*
	
	Electronic	Hybrid	Electronic	Hybrid	Electronic	Hybrid
**Equipment Costs** ***Equipment Costs***^***u***^	$33,404 *$35,582*	$4,835 *$5,204*	$157,930 *$167,910*	$29,915 *$21,443*	$279,492 *$295,819*	$38,056 *$41,742*

**Staffing Costs** ***Staffing Costs***^***u***^	$159,155 *$175.051*	$189,792 *$208,748*	$795,773 $875,254	$948,960 *$1,043,743*	$1,591,545 *$1,750,509*	$1,897,920 *$2,087,485*

**TOTAL COSTS** **(per capita costs)** ***TOTAL COSTS***^***u***^ ***(per capita costs)^u^***	$192,559 ($1.93) *$210,633* *($2.11)*	$194,627 ($1.95) *$213,952* *($2.14)*	$953,703 ($1.91) *$1,043,164* *($2.09)*	$978,875 ($1.94) *$1,065,186* *($2.13)*	$1,871,037 ($1.87) *$2,046,328* *($2.05)*	$1,935,976 ($1.94) *$2,129,227* *($2.13)*

**System Cost Difference** ***System Cost Difference***^***u***^		**$2,068** ***$3,319***		**$14,858** ***$22,022***		**$64,939** ***$82,899***

*Total costs have been discounted at 5%.

^u ^= Undiscounted

### Sensitivity Analysis

Deterministic one-way sensitivity analysis was performed on various model parameters for both scenarios. For several parameters, variation led to changes in the magnitude and/or direction of the total cost differences between electronic and hybrid systems.

#### Scenario 1 (1^st ^year pandemic + 4 years of seasonal influenza vaccination campaigns)

**Figure 1:**
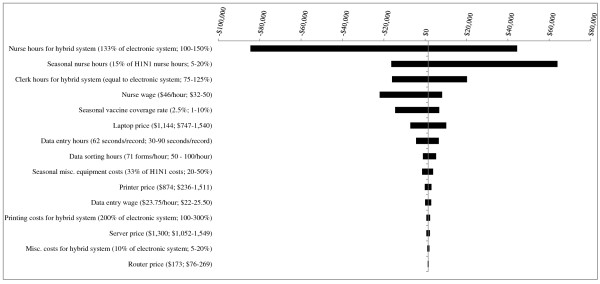
**Results of Sensitivity Analyses - Scenario 1 (1^st ^year pandemic + 4 years of seasonal influenza vaccination campaigns) for PHU population of 100,000**. The base case value and tested range is provided for each parameter. In the base case, the electronic system is less costly than the hybrid system, by a difference of $1,476. Where bars correspond to negative dollar values, variation in the parameter has resulted in the hybrid system becoming less costly than the electronic system.

**Figure 2:**
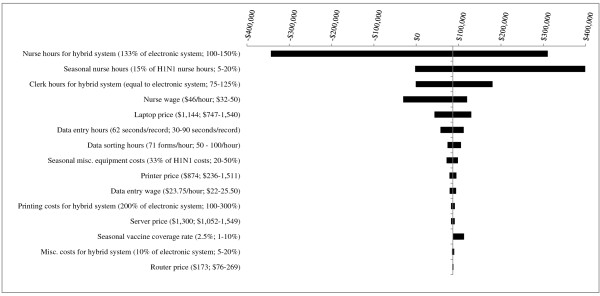
**Results of Sensitivity Analyses - Scenario 1 (1^st ^year pandemic + 4 years of seasonal influenza vaccination campaigns) for PHU population of 500,000**. The base case value and tested range is provided for each parameter. In the base case, the hybrid system is more costly than the electronic system, by a difference of $86,473. Where bars correspond to negative dollar values, variation in the parameter has resulted in the hybrid system becoming less costly than the electronic system.

**Figure 3:**
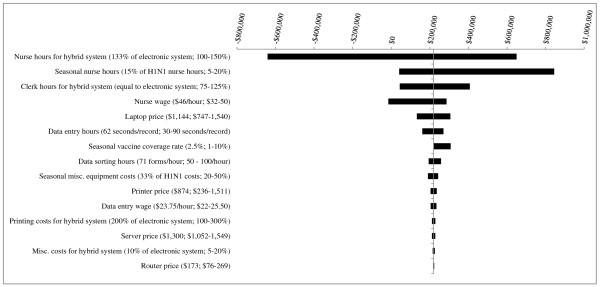
**Results of Sensitivity Analyses - Scenario 1 (1^st ^year pandemic + 4 years of seasonal influenza vaccination campaigns) for PHU population of 1,000,000**. The base case value and tested range is provided for each parameter. In the base case, the hybrid system is more costly than the electronic system, by a difference of $219,315. Where bars correspond to negative dollar values, variation in the parameter has resulted in the hybrid system becoming less costly than the electronic system.

For each PHU population size, several parameter changes resulted in the hybrid system becoming less expensive than the electronic system by fairly small margins (Figures 1, 2 and 3). One parameter change - decreasing *nurse hours for the hybrid system *from 133% to 100% that of the electronic system - led to a costing difference of greater than $50,000. For the smallest, medium-sized and largest PHUs, the between-system cost differences would be $84,533, $343,575, and $649,365, respectively.

Results from our threshold analysis demonstrated that in order for the electronic system to become less costly than the hybrid system, the total five-year software fees for small, medium and large-sized PHUs would have to increase to approximately $29,000, $143,000 and $281,000, respectively.

#### Scenario 2 (5 years of seasonal influenza vaccination campaigns)

**Figure 4:**
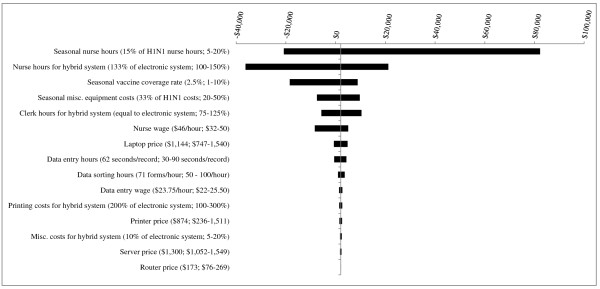
**Results of Sensitivity Analyses - Scenario 2 (5 years of seasonal influenza vaccination campaigns) for PHU population of 100,000**. The base case value and tested range is provided for each parameter. In the base case, the electronic system is less costly than the hybrid system, by a difference of $2,068. Where bars correspond to negative dollar values, variation in the parameter has resulted in the hybrid system becoming less costly than the electronic system.

For PHUs with a population of 100,000 (Figure 4), several parameter changes including decreasing *nurse hours during the seasonal influenza campaign *to 5% that of the H1N1 campaign and decreasing *seasonal vaccine coverage rate *to 1% led to the electronic system becoming more costly than the hybrid system. Decreasing *nurse hours for the hybrid system *from 133% to 100% that of the electronic system resulted in the largest costing difference, with the electronic system becoming $36,217 more costly than the hybrid system.

**Figure 5:**
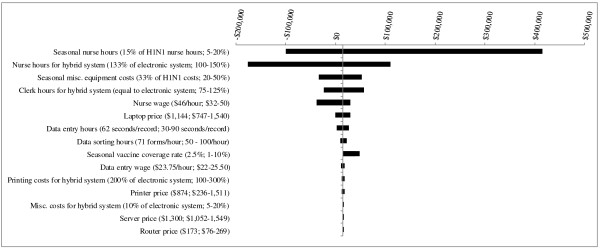
**Results of Sensitivity Analyses - Scenario 2 (5 years of seasonal influenza vaccination campaigns) for PHU population of 500,000**. The base case value and tested range is provided for each parameter. In the base case, the hybrid system is more costly than the electronic system, by a difference of $14,858. Where bars correspond to negative dollar values, variation in the parameter has resulted in the hybrid system becoming less costly than the electronic system.

For mid-sized PHUs (population = 500,000, Figure 5), two parameter changes led to the hybrid system becoming less costly than the electronic system by a difference of greater than $50,000: decreasing *nurse hours for the hybrid system *to be on par with that for the electronic system (between-system cost difference = $176,571); and decreasing *nurse hours during the seasonal influenza campaign *to 5% that of the H1N1 campaign (difference = $99,656).

**Figure 6:**
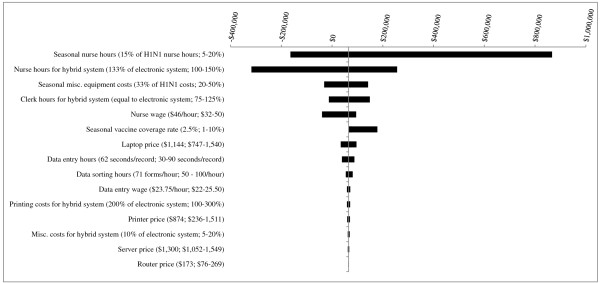
**Results of Sensitivity Analyses - Scenario 2 (5 years of seasonal influenza vaccination campaigns) for PHU population of 1,000,000**. The base case value and tested range is provided for each parameter. In the base case, the hybrid system is more costly than the electronic system, by a difference of $64,939. Where bars correspond to negative dollar values, variation in the parameter has resulted in the hybrid system becoming less costly than the electronic system.

For PHUs of population 1,000,000 (Figure 6), the same two parameter changes resulted in the hybrid system becoming less costly than the electronic system, but by larger margins: decreasing *nurse hours for the hybrid system *to be on par with that for the electronic system (between-system cost difference = $319,917); and decreasing *nurse hours during the seasonal influenza campaign *to 5% that of the H1N1 campaign (difference = $164,088).

From the threshold analysis, we found that the total five-year software fee costs would need to rise to $15,000, $73,000 and $140,000, for small, medium and large-sized PHUs, respectively, in order for the electronic system to be greater in cost than the hybrid system.

## Discussion

Based on our provincial model, the electronic immunization data collection system is not as costly when compared to the hybrid system as previously assumed, particularly when implemented during a pandemic vaccination campaign and/or used in medium- to large-sized PHUs. Our model's five-year projections indicate that the electronic system is less expensive than the hybrid system for all PHUs for both scenarios, and the difference in overall costs increases for larger population sizes.

The perception of high electronic system costs compared to more traditional approaches to immunization data collection is likely the result of evaluating implementation-year costs only. We examined these annual cost projections with our model: in a year when equipment is purchased for a pandemic campaign, the costs of the electronic system are 3% less costly than the hybrid system for medium-sized and large PHUs but higher than the hybrid system for the smallest PHU size ($291,595 vs. $283,300). Assuming equipment is purchased for a regular influenza vaccination campaign, annual electronic system costs are 17-19% more expensive than hybrid system costs for every PHU population size. However, as laptops/routers/servers can typically be used for five campaigns before requiring replacement, a five-year time-frame offers a more appropriate data collection system cost comparison.

Given that mass immunization clinics require extensive human resources, including nurses, registration clerks, data entry clerks and IT support staff, staffing will be a main contributor to the total costs for all immunization data collection systems, regardless of whether they are fully electronic or paper-based approaches. Although our model is based on a single proprietary solution, we have demonstrated how important it is for public health agencies to consider all available options and not underestimate the cost-savings attributed to the reduction in staffing hours that are associated with electronic data collection. Our study's results can be applied to other fully electronic data collection systems which may be employed during future large-scale vaccination campaigns: as exemplified by our threshold analysis, unless an electronic system's software fees are prohibitively high, the increased equipment expense is unlikely to negate the savings in costs associated with moving from a hybrid to an electronic system, thereby reducing nurse hours and eliminating the need for data entry clerks. Additionally, at least a portion of the equipment required, including laptops and servers, can be used for other programs within the same jurisdiction, thereby decreasing overall costs to the agency.

Sensitivity analyses indicated that results were more affected by changes to several staff-related variables than equipment-related changes, which is to be expected as the bulk of the total system costs are related to staffing rather than equipment. Results were sensitive to our assumption that the hybrid system required 33% more nurse hours than the electronic system. We conducted an additional analysis and found that should the hybrid system require at least 26% more nurse hours than the electronic system, the electronic system will be approximately equal in cost or less expensive than the hybrid system for medium and large-sized PHUs.

During the pandemic campaign, clinics employing hybrid systems often implemented approaches to reduce staff workload and consequently, related costs, which could also be used during seasonal influenza vaccination campaigns: having clients complete their own consent forms could potentially reduce the amount of clerk resources required at registration [5]. However, this process requires clients to be knowledgeable about their medical history, and could add time to the post-vaccination data entry stage should forms be illegible or incomplete.

Several limitations should be considered when interpreting the results of this study. Many of the assumptions regarding the cost and resources used for the H1N1 campaign (Year 1) were based on a panel of six Ontario PHUs. Because each of these agencies had previously used hybrid systems for their seasonal influenza vaccination campaigns, we are confident in the validity of their responses. However, as participating PHUs provided itemized costs for the pandemic campaign only, there is some degree of uncertainty around the seasonal influenza resource assumptions on which the model was developed, heightening the importance of the sensitivity analyses.

There are potential limitations to this study based on staff-related assumptions. While hybrid systems may require minimal IT support to set up the data entry databases, no estimates were given of this time so it was excluded from the model; however, inclusion would not be expected to significantly increase hybrid system costs. It is also possible that jurisdictions using hybrid systems during seasonal campaigns may decide to forgo the post-vaccination data entry step and instead retain client data in paper form, restricting the ability to use the data but eliminating the costs of data entry clerks.

A related limitation extends to the electronic system: typically, some data cleaning is required before the information is in a usable state. This time was not consistently included in clerk time estimates from sources, meaning that resources required for an electronic system may be slighter higher than was reported. However, it is assumed that this effect is more pronounced in the first year of the system's use in a jurisdiction, and diminishes with time, as the system is modified by developers to enhance usability. A further limitation of this study is that an electronic system failure was not modeled; depending on the length of such an event, organizations using the electronic system could either wait until it was resolved, or revert to the hybrid system in the interim. Both scenarios could result in increased staff time, and consequently, increased staff costs. To safeguard their clinics against many technical issues, it is common practice for organizations to purchase a backup server (as did the majority of PHUs who provided us with data for this study).

Only the costs incurred related to the immunization data collection system in use were included in this evaluation. Benefits attributed to the implementation of each system were not measured, but are numerous: rapidly available data to ascertain coverage rates overall as well as by certain risk groups; the contact of individuals should vaccine requirements change; and quick retrieval of records by vaccine lot number to determine whether certain vaccine batches are linked to higher rates of adverse events. Previous studies have attempted to measure the monetary values associated with the development of vaccine coverage reports, the quick assessment of a client's immunization history and other capabilities of an electronic system [7]. This type of measurement is challenging, although its inclusion in this study would have decreased the overall costs of the electronic system.

Finally, the ability to apply the results of this evaluation to other jurisdictions is dependent on the specifics of the electronic and hybrid systems they are using. Our findings may not be applicable to jurisdictions that are considering immunization data collection programs that vary considerably from PECS in terms of factors most influential to cost such as training time and staff hours required. Even if less nurse and data entry clerk hours are needed for the electronic system being compared, the overall monetary savings to the PHU may only be measurable if staff are not paid during the newly free hours. Should staff be assigned to different activities during their time saved, the total costs to the unit would not change although the costs related to the vaccination campaign would decrease.

Despite these limitations, this economic evaluation is the first Canadian costing evaluation of immunization data collection systems and makes a valuable contribution to overall electronic and hybrid system comparisons. Several Canadian provinces and territories currently have their own immunization registries, but these are not/cannot be used at the point of vaccination, and therefore client-level immunization data are collected on paper forms and transferred into the registry at a later time; it is possible that fully electronic systems, like PECS, could be integrated into these registries in the future, to eliminate the post-vaccination data entry step that is currently required. Additionally, as Panorama, a public health surveillance system with a planned immunization registry module, is implemented in several Canadian provinces and territories over the next several years, the need for a separate automated immunization system in these jurisdictions may lessen. However, because not every province and territory is planning to adopt Panorama, it is likely that standalone electronic immunization systems may still be of value to many Canadian public health organizations. This study may also have implications for vaccination programs in other developed countries in which at least a portion of vaccines are delivered by public health.

## Conclusions

Although the cost of electronic systems has been previously identified as a major barrier to their implementation, results of this study indicate that over a five-year projection, the costs of hybrid and electronic systems may be very comparable. Collecting client-level data in electronic form is useful for numerous reasons such as monitoring coverage at various levels of geographical granularity in real-time, directing health promotional activities, and surveillance of vaccine safety and efficacy. The value of such benefits should be recognized by public health organizations when planning for future influenza seasons.

## List of Abbreviations

IT: information technology; PECS: Protocol for Electronic Clinic Systems; PHU: public health unit

## Competing interests

The authors declare that they have no competing interests.

## Authors' contributions

All authors participated in the conception and design of this economic evaluation. JP, JF, CH and SQ participated in data collection. JP, JF and BS were involved with model development. All authors were involved in either drafting the manuscript or providing revisions, and each has given final approval of the version to be published.

## Pre-publication history

The pre-publication history for this paper can be accessed here:

http://www.biomedcentral.com/1472-6963/11/210/prepub

## References

[B1] Writing team for the Public Health Agency of Canada/Canadian Institutes of Health Research Influenza Research Network Vaccine Coverage Theme Group Why collect individual-level vaccination data? CMAJ 2010 182 3 273 75 20048007 10.1503/cmaj.091515 PMC2826469

[B2] Heidebrecht CL Foisy J Pereira JA Quan SD Willison DJ Deeks SL Finkelstein M Crowcroft NS Buckeridge DL Guay M Sikora CA Kwong JC Perceptions of Immunization Information Systems for Collecting Pandemic H1N1 Immunization Data within Canada's Public Health Community: A Qualitative Study BMC Public Health 2010 10 523 10.1186/1471-2458-10-523 20807421 PMC2941494

[B3] Canadian Agency for Drugs and Technologies in Health Guidelines for the Economic Evaluation of Health Technologies 2006 http://www.cadth.ca/media/pdf/186_EconomicGuidelines_e.pdf Accessed Dec 21, 2010

[B4] Pereira JA Quach S Heidebrecht CL Foisy J Quan SD Finkelstein M Sikora CA Bettinger JA Buckeridge DL McCarthy A Deeks S Kwong JC Pan-Canadian assessment of pandemic immunization data collection: study methodology BMC Medical Research Methodology 2010 10 51 20624270 10.1186/1471-2288-10-51 PMC2896946

[B5] Quach S Hamid JS Pereira JA Heidebrecht CL Foisy J Bettinger JA Rosella L Crowcroft NS Deeks SL Quan SD Finkelstein M Guay M Buckeridge DL Sikora CA Kwong JC Time and motion study to compare electronic and hybrid data collection systems during the pandemic (H1N1) 2009 influenza vaccination campaign Vaccine 2011 29 10 1997 2003 10.1016/j.vaccine.2010.09.016 20863900

[B6] Billittier AJ Lupiani P Masterson G Masterson T Zak C Electronic Patient Registration and Tracking at Mass Vaccination Clinics: A Clinical Study J Public Health Management Practice 2003 9 5 400 10 10.1097/00124784-200309000-00011 15503605

[B7] McKenna VB Sager A Gunn JE Tormey P Barry MA Immunization Registries: Costs and Savings Public Health Reports 2002 117 386 9 12477921 10.1016/S0033-3549(04)50176-0 PMC1497442

